# What Should Personalised Mental Health Support Involve? Views of Young People with Lived Experience and Professionals from Eight Countries

**DOI:** 10.1007/s10488-024-01382-2

**Published:** 2024-06-22

**Authors:** Ayesha Sheikh, Jenna Jacob, Panos Vostanis, Florence Ruby, Inga Spuerck, Milos Stankovic, Nicholas Morgan, Catarina Pinheiro Mota, Rúben Ferreira, Şeyda Eruyar, Elmas Aybike Yılmaz, Syeda Zeenat Fatima, Julian Edbrooke-Childs

**Affiliations:** 1 Anna Freud, 4-8 Rodney Street, London, N1 9JH UK; 2 Euro Youth Mental Health, The Carling Building, Coopers Yard, Off, Market Pl, Hitchin, SG5 1AR UK; 3 Department of Psychology, Necmettin Erbakan University, Köyceğiz, Meram, Konya, 42140 Turkey; 4 School of Media, Communication and Sociology, University of Leicester, University Road, Leicester, UK; 5 Clinical, Educational and Health Psychology, University College London, London, UK; 6 University of Trás-Os-Montes and Alto Douro, Vila Real, Portugal; 7 Center for Psychology, University of Porto, Porto, Portugal; 8 Hussaini Foundation-Child and Adolescent Development Program, Karachi, Pakistan

**Keywords:** Young people’s mental health, Mental health support, Mechanisms, Participatory action research, Personalised care

## Abstract

Research demonstrates that young people value mental health support that is tailored to their needs and preferences, rather than a “one size fits all” offer, which is often not equitably accessible (National Children’s Bureau, 2021). Understanding young people’s lived experiences across different sociocultural contexts is important. The aim of this research was to conduct an international qualitative study on the views of young people with lived experience and professionals, on proposed aspects of personalised support for anxiety and/or depression. Participatory action focus groups were conducted with *N* = 120 young people with lived experience of anxiety and/or depression (14–24 years) and with *N* = 63 professionals in Brazil, India, Kenya, Pakistan, Portugal, South Africa, Turkey, and the United Kingdom. Data were analysed using the rigorous and accelerated data reduction (RADaR) technique. Overall, although some country-specific differences were found in terms of what aspects of support young people found to be most important, individual preferences were considered stronger, furthering the view that support should be personalised to the needs of the individual young person. Young people experiencing anxiety and/or depression should be able to choose for themselves which aspects of support they would prefer in their own care and support plans, with families and mental health professionals providing guidance where appropriate, rather than removing the young person from the decision-making process altogether. It should also be ensured that the aspects of personalised support can be understood by young people and professionals from different contexts, including marginalised and minoritised groups and communities.

Worldwide, mental health difficulties are of significant concern, with one in ten young people experiencing anxiety and/or depression (GBD, 2017). There is evidence that mental health difficulties in young people are increasing (Edbrooke-Childs et al., 2017; GBD, 2017), and have been further exacerbated by the COVID-19 pandemic (Singh et al., 2020). Nevertheless, evidence suggests that the majority of young people experiencing mental health difficulties will not receive a specialist mental health intervention (Belfer, 2008). For example, research shows that only around 18–34% of young people experiencing high levels of anxiety and depression seek professional help (Gulliver et al., 2010). Further, many young people will experience pressures on their mental health, for example from social media use and online bullying, worries about climate change, and loneliness, that do not meet the threshold for a clinical diagnosis of a named condition, but still have a significant impact on their daily life and wellbeing (Goodfellow et al., 2022; O’Reilly et al., 2018a, b; Ma et al., 2022; Suh & Lee, 2023). The scale of the youth mental crisis can therefore be obscured as difficulties such as these can go “unseen” and may not be included in official mental health statistics.

Where young people’s difficulties do reach thresholds for specialised support, there are huge inequalities in the allocation of mental health resources across regions and between low-middle-income countries (LMIC) and high-income countries (World Health Organization, 2020). This is despite the majority of young people worldwide living in LMIC (UNICEF, 2016), and the particular vulnerabilities young people in LMIC are exposed to, resulting in multiple interlinked disadvantages (Kieling et al., 2011). To maximise available resources, there is growing attention on approaches for prevention and intervention at the societal, community, family, and individual levels; for example, social prescribing (Bickerdike et al., 2017), community mental health care (Thornicroft et al., 2016), resilience building interventions in LMIC (Tamburrino et al., 2020), and school-based interventions (O’Reilly et al., 2018a, b). However, a key unanswered research question is what, for whom and under what circumstances do mechanisms of mental health support work for young people? Understanding what aspects of personalised support for the prevention and intervention of mental health difficulties are effective, forms the basis of being able to provide meaningful care.

Evidence from systematic reviews suggests that a range of cognitive, emotional, behavioural, relational, and systemic factors mediate the effects of prevention and intervention support for anxiety and/or depression (Stirling et al., 2015). For example, supportive, trusting, and caring relationships with informal and structural providers are consistently identified as a facilitator of change (O’Keeffe et al., 2020). However, more evidence on the core processes that lead to change when supporting mental health is urgently required to contextualise and understand these findings to ultimately improve support for young people (Wellcome Trust, 2021). It has been suggested that current models of mental health care do not meet the needs of most young people, and that measurement-based personalised mental health support could help to inform clinical decisions as this would consider many of the aforementioned individual factors (Iorfino et al., 2022).

The concept of personalised support, or tailoring interventions, for individual needs and preferences, has been gaining popularity in the field of youth mental health care in the last few years, demonstrated for example, by its inclusion in the National Health Service (NHS) Long Term Plan in the UK (NHS England, 2019), and National Health Strategies in Australia (National Mental Health Commission, 2021). Personalised support allows clinicians and clients to collaborate on creating goals and to co-produce a plan for working together. This way of working can often be seen as more acceptable to those receiving support, as it allows for important flexibility (Bennett & Shafran, 2023; Coulter et al., 2015). In the UK, the THRIVE framework (Wolpert et al., 2019) is an example of a model of support for young people based on a personalised clinical approach driven by an understanding of their needs at assessment. Elsewhere, in Australia, the Brain and Mind Centre (BMC) Youth Model has been developed to use personalised assessments and measurement-based outcomes to deliver highly personalised support and interventions for young people with various mental health needs (Hickie et al., 2019). This model has been designed for use with health information technology to further improve the accessibility and appeal of youth mental health care (Davenport et al., 2020). One review of UK-based personalised approaches to community youth mental health care found there was a positive impact on the young people who received them, as they valued how support was tailored to their own needs and preferences and not a “one size fits all” offer, which was often not accessible anyway (National Children’s Bureau, 2021). Previous research has recognised that adopting an effective person-centred approach is often more challenging in LMICs, however, it can still be achievable with careful planning and development of the healthcare service, with a framework for LMICs to transition to person-centred care available (Mahendradhata et al., 2014).

It has also been proposed that highly personalised support can improve stepped-care approaches for young people by considering the complexity of their needs, which can involve measurement-based outcomes based on what is important to the young person, rather than just the clinician (Cross et al., 2019; Jacob et al., 2017; Krause et al., 2019). However, more research is needed into precisely in which ways personalised support can best help young people with anxiety and/or depression, and be effectively incorporated into routine practice, particularly on an international level (Iorfino et al., 2022; Jahedi et al., 2024; Ng & Weisz, 2016).

Moreover, there is increasing concern about the extant literature underrepresenting young people from minoritised and marginalised groups, particularly in relation to ethnicity and race, and those from LMIC (Razzouk et al., 2010). In LMIC, young people’s access to formal mental health services is negatively impacted by stigma, living in adversity, and lack of specialist resources, thus they are more likely to access informal community support (Getanda et al., 2017; Patel et al., 2018; Vostanis et al., 2022). This underlines the importance of understanding how young people view personalised support across different sociocultural and service systems.

The present research aims to address these research gaps and to explore the views of young people with lived experience, and professionals internationally, regarding what effective, personalised support might look like for them in the prevention and treatment of anxiety and/or depression, which are the most common mental health difficulties worldwide (GBD 2019 Mental Disorders Collaborators, 2022). Specifically, the research questions are: “what aspects of personalised support do young people with lived experience and professionals view as effective for young people experiencing anxiety and/or depression?” and “are there country-specific differences in the aspects of personalised support that young people with lived experience and professionals view as effective for young people experiencing anxiety and/or depression?”.

## Methods

### Study Design

While the focus of this research is primarily on anxiety and depression, personalised support is more wide-reaching, particularly concerning prevention and early intervention, such that this includes mental health and wellbeing difficulties – and wellness – in addition to and often as precursors to diagnosable mental health difficulties. The focus on anxiety and depression for this research was due to the recognition that anxiety in particular has an earlier onset than other mental health difficulties (Solmi et al., 2022) and tends to develop in tandem with other mental health difficulties. As such, it has been identified as a key target of focus by the funders of this research: the Wellcome Trust.

Health research increasingly values the importance of involving patients, clients and members of the public in order to work with them, rather than develop research on them. This has been further encouraged internationally through the integration of patient and public involvement (PPI) increasing recognition of this participation in health-care research (McCoy et al., 2019; Wilson et al., 2015). The integration of PPI, particularly young people, represents a new way of creating science in the health domain, and the success of PPI on research often relies on the nature of the interactions between individuals involved in the process. PPI also has a relevant implication on final reports that benefit from being grounded in user experiences, by providing a wider, more relevant viewpoint, ensuring cultural relevance, and enhancing the credibility of findings with stakeholders (Brett et al., 2014).

Participatory Action Research (PAR; (Baum et al., 2006)) often involves collaboration between researchers and the community with lived experience, and is increasingly used to address issues affecting individuals who are marginalised or usually excluded from service planning such as young people (Rhodes et al., 2012). PAR was considered most appropriate for the present research, as it centralises young people and their voices in the design, delivery and evaluation of the project, which is fundamental to the research aim. Overall, a qualitative design was appropriate for this research to give depth and context to the exploration of the views of young people and professionals, which might otherwise not be possible.

### Participants

Participants comprised 120 young people (84 female, 36 male) and 63 professionals (37 female, 26 male), who were recruited through local and online advertisements in eight partner collaborating countries, representing a wide socioeconomic spectrum (OECD), 2016): Kenya, Pakistan, India, Brazil, South Africa, Turkey, Portugal and the United Kingdom. The particular countries were included because of established partnerships with the research team (Vostanis et al., 2019). There was also an emphasis from the funder on including LMIC in this research. Within each country, a non-governmental organisation (NGO – Brazil, India, Kenya, Pakistan, South Africa and UK) or academic institution (Portugal and Turkey) acted as local project lead. These lead agencies were identified through existing international youth mental health networks by the research team (World Awareness for Children in Trauma; Child Outcomes Research Consortium). The project leads in each country co-ordinated participant recruitment, to the eligibility criteria and to their preferred way of engaging with their local communities.

Young people were eligible to participate if they were aged between 14 to 24 years and had self-identified lived experience of anxiety and/or depression. The age range is that which is of focus to the study funder, and while the term young people is used, the population may also be conceived of as emerging adults. This is a critical period in development, when anxiety and depression are often prevalent, not least due to the instability, self-focus and feelings of being in-between childhood and adulthood that are often present at this time (Arnett et al., 2014). Anxiety and depression in this study were defined as difficulties and disorders as defined in published literature, inclusive of various types of anxiety, e.g., general and specific anxiety, panic disorders, obsessive–compulsive disorder and post-traumatic stress disorder, and clinical low mood and major depressive disorder. Professionals were recruited if they worked in the youth mental health field; participants mainly comprised mental health and non-specialist professionals, and researchers. Sample size was determined through a combination of convenience sampling and prior experience and knowledge about the most effective number of participants and focus groups required in research (a minimum of five per group; a minimum of four groups; see (Cortini et al., 2019; Hennink et al., 2019).

Youth participants were supported throughout their involvement in the study by Peer Advisory Groups, which were provided by Euro Youth Mental Health as a regular form of peer support and a way to engage young people across the participating countries to share their experiences and feedback. The advisory group meetings took place every few months throughout the project.

### Research Procedure

Ethical approval for the study was obtained from the Psychology Research Ethics Committee at the University of Leicester (approval number 22748). Lead agencies acted as gatekeepers to the study, according to local ethics jurisdictions. All participants gave written informed consent prior to taking part in the study. Consent was sought from parents and carers of young people aged 14 to 15 years, in which case verbal assent was also obtained from the young people.

Young people with lived experience of depression and/or anxiety were also involved as peer advisors throughout the project to ensure that the research activities and outputs were in line with young people’s understanding and views, as well as compatible with the sociocultural norms of each country/site. Each local agency recruited one peer advisor, as an expert in their own community, to co-deliver focus groups, effectively engage young people from their country, ensure that communications and materials were tailored to young people and reflected country-specific considerations, and ensure that the interpretation of the data reflected young people’s perspectives.

Disagreements that arose in the discussions were explored at the time of arising, in what is known as “communicative action” (Habermas, 1996; Kemmis, 2006) and presented in the data for analysis. Communicative action refers to the deliberate process of acknowledging differing interpretations of a concept or situation, and the participants in the discussion engaging in self-expression to arrive at an agreed way forward. The analysis was conducted by several researchers, including the peer researchers, to ensure priority was given to the young people’s voices in the analysis. This recognised the need to explore and value the different types of knowledge, experience and insight, in a “mutual research relationship” (Newton & Parfitt, 2013).

As well as leading on the youth engagement, Euro Youth Mental Health provided two Peer Researchers from Germany and Serbia, who were centrally involved throughout the project as part of the research team. The Peer Researchers played a key role in project governance through all elements of the research, including co-designing materials, co-facilitating focus groups, and contributing to the analysis and write up.

### Data Collection Process

In each country, the local organisation facilitated two focus groups with young people (range between 12 and 23 participants) and one focus group with professionals (range between 6 and 10 participants). Each focus group was co-facilitated by a peer advisor and a professional lead based in the country. Focus groups were either facilitated face to face (Turkey, South Africa, India, Kenya) with the research team joining remotely, or online via a digital platform (Brazil, UK, Pakistan, Portugal) depending on the country’s safety guidelines regarding the COVID-19 pandemic. Following local focus groups, two cross-country focus groups were facilitated with all local partners, separately for young people and professionals, to review emerging findings. The purpose of the cross-country focus groups was to check, refine and explore the emerging findings in relation to different country contexts. This validation step ensured that the understanding and presentation of the findings bolstered the international focus of the research and ensured that weight is not given unduly to some countries over others.

A semi-structured focus group schedule was used to ask open-ended questions regarding participants’ views of 26 aspects of personalised support, or ‘active ingredients’ (Wellcome Trust, 2021) of support, for young people experiencing anxiety and/or depression. The list had previously been compiled by the study funder, commensurate with an overarching research strategy whereby independent research teams proposed and researched individual elements, or aspects, of personalised support (see (Wellcome Trust, 2021)). Participants were prompted to discuss the following topics: how their cultural context impacted on the relevance of the aspects of personalised support discussed, whether anything needed to be reworded, removed from, or added to the list, and their views regarding what they considered most and least helpful in their lived experiences of anxiety and/or depression, or clinical/research work. In some focus groups (young people: Brazil, Kenya, Pakistan, South Africa; professionals: Brazil, Kenya, Turkey, UK) participants also discussed how the list could be better organised, grouped or merged, and provided suggestions of overarching themes.

Each focus group lasted between two to three hours. Focus groups were audio-recorded and conducted in the local language, except for the cross-country focus groups which were conducted in English. The audio recordings were transcribed and translated into English before analysis.

### Data Analysis

The Rigorous and Accelerated Data Reduction (RADaR) technique was used to code and analyse the data (Watkins, 2017). This technique was employed to allow for the rapid analysis of a large quantity of qualitative data over a short period of time. An Excel spreadsheet was created with each column representing one topic of the focus group guide, and each row containing one excerpt from the data that provided information about the specific topic of the focus group guide. The data were organised across the following four columns, which sought to mitigate some of the identified risks relating to the use of a predefined list of aspects of personalised support (particularly columns a and c): a) Views on the presented list (things to add, unclear meaning, combining/ separating aspects of personalised support); b) Helpful and unhelpful aspects of personalised support, strengths and limitations; c) Changes to the organisation of the list; and d) Individual context and systemic factors influencing aspects of personalised support. An additional column was included to summarise each transcript excerpt. Individual summaries were then further combined to provide information regarding each area of the focus group topic guide.

One researcher (FR) coded transcripts from six countries while two peer researchers (IS, MS) coded transcripts from two countries. Following the completion of all coding, the research team compiled a revised list (including all original, edited and newly added ones) and combined those that were identical, highly related or provided examples of a specific aspect of personalised support. This process led to the creation of a single, cross-country list of 65 aspects of personalised support which was then organised following an inductive thematic approach (Braun & Clarke, 2019, 2021). The derived themes were generated by the research team following an iterative approach, and were reviewed and updated based on feedback from young people (N = 17) and professionals (N = 11) during two cross-country focus groups. A senior researcher (JEC) cross-checked the original coding during the compilation and organisation into themes stages. The final list of themes was then organised following Bronfenbrenner’s ecological systems theory (Bronfenbrenner, 1992), which focuses on different levels of the environments in which a child grows up in and the impact of these on their development. The environments vary from microsystems at the person level, to macrosystems comprising the wider society and environmental factors. Therefore, the findings are organised with themes over which young people could have more control over (e.g., “Understanding and accepting yourself”) located closer to the centre, and themes over which young people have less control (e.g., “Society and the environment”) located further away from the centre.

### Methodological Reflexivity

The researchers were mindful of potential tensions between the deductive nature of using the original list of aspects of personalised mental health support, and the overarching areas of the focus group topic guide in the RADaR analysis, and the inductive nature of the subsequent thematic analysis, which realises researcher subjectivity to be a resource which aids the generation of themes. In each country, focus groups were thus conducted by local partners and peer advisors to ensure that discussions were informed by contextual knowledge rather than potentially influenced by the research team’s Europe-based backgrounds and experiences. Data analysis was carried out solely by University-educated, Europe-based researchers. While it may not be possible to erase the power dynamics that exist in traditional research or academic sectors, our research went some way to mitigate these through the involvement of the peer researchers and the peer advisors. Involving the peer researchers as ingrained members of the research team ensures they are not considered separate advisors or consultants. In addition, the research involved peer advisors as well as peer researchers, who led the liaison with participants. This aimed to bridge the gap between the academic researchers and the participants, however, this is likely to have been experienced differently in different countries. The research process primarily involved relationship building between the peer researchers and peer advisors as opposed to academic researchers in the team. While there are wide-ranging lived experiences amongst the researchers (of mental health difficulties, being a parent, being a young person), these educational and cultural factors will have had an impact on how the research was delivered and the findings understood. Nevertheless we aimed to consider a wide range of experiences when compiling the final list and generating themes. The role of peer researchers was particularly important at this stage, as they were able to reflect on their experiences (therefore considering what other young people might think, which is key when conducting research with young people). They also ensured that the final list was inclusive and accessible to a wide range of young people (e.g., considering the wording and how concepts could be easily translated from English into other languages). Throughout the data analysis, feedback from peer advisors and local partners was sought to ensure that emerging findings reflected the views of participants who engaged in focus groups, and to reduce biases towards the experiences of participants in one country over others.

## Findings

**Fig. 1:**
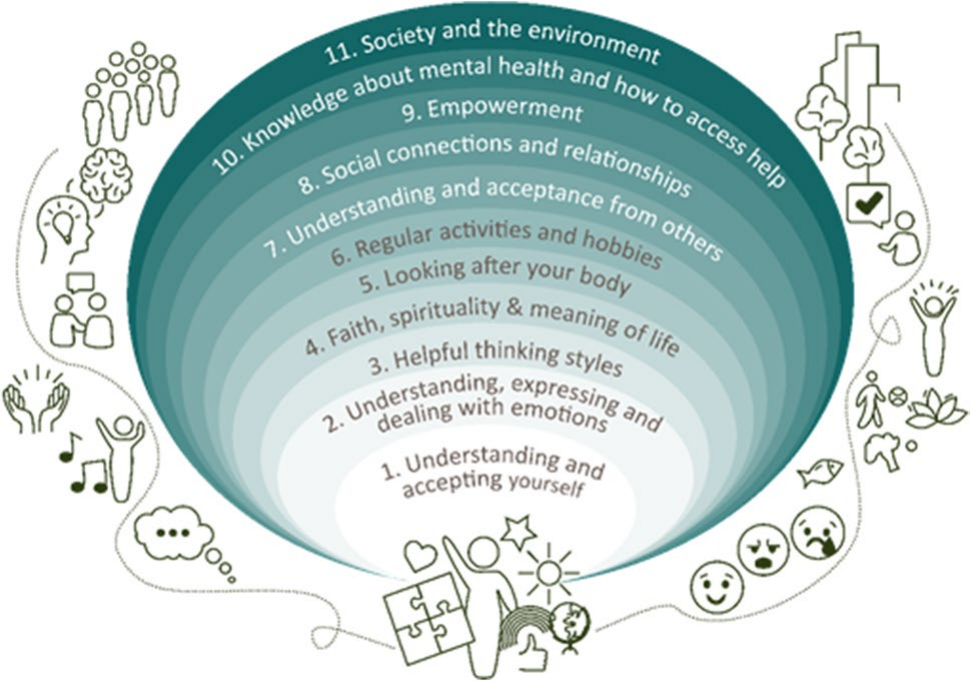
Themes according to the socioecological system framework

Sixty-five aspects related to personalised support were agreed across groups, which were organised into eleven themes and are presented according to the socioecological systems framework in Fig. 1. A full list of themes and aspects of personalised support is presented in Table 1. The findings were organised according to a description of the cross-country findings, followed by three main subheadings which broadly represent a socioecological framework: micro, meso, and macro levels, and are linked to prior research where possible. All provided quotes are from participants from the young people's groups. The findings have been presented this way to promote the applicability and practicality of the findings in everyday life and mental health support. The juxtaposition between young people and professionals’ perspectives relating to the wider support systems was not within the scope of this current research, and is discussed in detail elsewhere (Vostanis et al., 2022).

**Table 1:** Themes and corresponding active ingredients

Themes	Active ingredients
Understanding and accepting yourself The process of focusing on demonstrating kindness to yourself, of accepting who you are and what difficulties you are experiencing. This includes young people working on how they talk to themselves and perceive themselves.	Acknowledging and accepting how things are (e.g., difficulties you are experiencing)
	Being kind and respectful to yourself (e.g., reduced self-criticism)*
	Developing ability to be independent and make decisions
	Improved view of self and self-confidence (e.g., body image)*
	Taking time to learn about and understand yourself
Understanding, expressing and dealing with emotions Linked to understanding and accepting yourself, there is a focus here on young people understanding and accepting their own emotions. This also includes working on both expressing their emotions, and regulating and managing their emotions. This might be through learning new techniques and strategies to manage stress.	Expressing emotions
	Regulating and managing emotions*
	Understanding and accepting emotions
	Using relaxation techniques to manage stress*
Helpful thinking styles A focus on facilitative and helpful thinking styles. This includes learning new skills and techniques relating to thinking patterns, facing fears and learning problem solving skills. There is also a managing and reducing element to this, relating to managing perfectionism and negative thinking patterns.	Ability to shift perspective (e.g., take flexible views about yourself)*
	Developing new thinking patterns and understanding thinking patterns better*
	Developing the ability to manage and face your fears*
	Helpful use of mental imagery*
	Learning problem solving skills*
	Learning to manage perfectionism*
	Reduced negative thinking (e.g., thinking about the worse-case scenario)*
Faith, spirituality & meaning of life Higher order elements of personalised support related to developing or finding a sense of purpose, practising a religious or spiritual belief and feeling connected to a higher power.	Ability to be optimistic and hopeful about the future (e.g., goal-setting) *
	Being connected to a higher power
	Practising religious or spiritual belief
	Search for meaning in life
	Sense of purpose
Looking after your body This includes young people looking after themselves physically, which encompasses good sleeping patterns, better diet and exercise patterns and working on addictions.	Better diet to keep the body in balance (e.g., healthy eating and drinking)*
	Better sleep and body clocks*
	Reducing addictions
	Reducing the body’s stress reaction*
	Regular physical activities (in healthy amounts)*
Regular activities and hobbies A focus on increasing activities that have a positive impact on mental health; keeping busy with positive activities and reducing negative activities. This includes a range of activities such as engaging with nature and creative activities.	Developing routines and making good use of free time or keeping busy
	Engaging less in activities that have a negative impact on us
	Engaging with activities that have a positive impact on us*
	Engaging with creative activities*
	Engaging with nature (e.g., walks in the park)
Understanding and acceptance from others This includes feeling understood and accepted by both friends and family, but also acceptance from professionals in helping professions. Further important facets are feeling understood and included in terms of who young people are, and feeling a sense of belonging and mattering to others.	Acceptance from professionals when seeking or receiving mental health support
	Feeling understood and accepted by family and friends
	Improved understanding and inclusion of diversity (e.g., race, disability, LGBTQIA + , neurodivergence)
	Increased sense of belonging (e.g., being recognised by others)
	Increased sense of mattering to others*
Social connections and relationships A focus on working on social connections and relationships. This includes young people engaging in activities with their local community, improving intergenerational cohesion across neighbourhoods and being kind and respectful to others, including offering themselves to support others. A focus on improving social skills and managing relationships, which includes engaging in positive support networks. It was considered that a focus on these areas would support a reduction in loneliness.	Being kind to and respectful of others (e.g., volunteering)
	Engaging with the community
	Improved social relationships and interpersonal skills*
	Increased neighbourhood cohesion (e.g., across generations)*
	Managing relationships (e.g., engaging with positive support networks)
	Meaningful social connections*
	Reduced loneliness*
	Relationships with family
Empowerment This includes access to people who will advocate and represent young people, as well as enabling young people to manage their own health. This might also be through feeling able to contribute to social or systemic change. Further, increased self efficacy; the belief that a young person has that they can change their circumstances.	Access to advocacy and representation
	Empowering young people to manage their health and the support received
	Feeling of being able to contribute to social or systemic change
Knowledge about mental health and how to access help A focus on gaining knowledge about mental health difficulties and how to access support that may be considered effective. This may also encompass empowering families to support young people, through the acquisition of knowledge. It also includes learning new strategies to cope. Understanding and accepting mental health support is also considered important here, as well as medication following professional advice.	Accessing support for processing trauma and grief
	Awareness and education about mental health and reducing stigma
	Empowering the family to support young people
	Learning and developing helpful coping strategies (and avoiding unhelpful ones)
	Medication (following professional advice) (e.g., anti-depressants, anxiolytics)*
	Receiving professional help (both evidence-based interventions and new approaches)
	Understanding and accepting mental health support
Society and the environment A focus on elements outside of young people that relate to the systems, structures, society and the environment around them. This includes accessing information about financial support and money management, as well as increased financial stability. In addition, better access to welfare, nature and feeling safe in their environment. having basic needs met, preventing and reducing trauma as well as reducing discrimination in society.	Access to a workforce that represents young people and their communities
	Access to welfare
	Accessing information for financial support and better management of money
	Better city access to nature*
	Feeling safe
	Having basic needs met (e.g., food)
	Increased availability of quality health services
	Increased communication between different institutions and services
	Increased financial resources and financial stability (e.g., cash transfer)*
	Preventing and reducing trauma
	Promoting equity and decreasing discrimination and social injustice

^*^ = aspects identified by researchers in the original list (wording may have been modified in the current research). All other aspects in the table were identified through the focus groups

### Cross-country

Overall, participants consistently agreed that the opportunity to tailor and personalise support to an individual young person’s needs was key to potential engagement and effectiveness. Participants described the process of tailoring aspects of personalised support from a long list as something that could be facilitated with support from a professional or by a young person alone. While there were some cross-cultural differences, and some concepts did not translate into local languages (e.g., neighbourhood cohesion, perfectionism and self-compassion), participants suggested that shorter country-specific lists would be insufficient due to the heterogeneity of contexts within countries, e.g., participants from Brazil recruited from as widely as the city of Sao Paolo and the Amazon region.

Further, cultural contexts led to variation in perceived relevance. For example, some aspects of personalised support were identified as less relevant within some contexts, such as neighbourhood cohesion (Brazil, Portugal), better urban access to green spaces (Brazil, Pakistan), and reduction of inflammation levels in the body (Portugal); consequently, there was little discussion generated. However, these were reflected as important amongst participants in other countries. In Turkey, for example, young people jointly talked about the stress and anxiety caused by the relationship between relatives and neighbours, e.g., “it can create pressure. This is common in our country: when we come home late, the neighbour intervenes, telling our parents […] This creates psychological pressure on us” (participant, Turkey). Social connections are expected to be beneficial, but interference in private life and disrespect for decisions can cause negative emotions. In Turkey and Pakistan, young people said that parents were often the ones deciding if or when a young person should seek professional help. In Kenya and India, young people reported a strong stigma associated with the use of antidepressants, which were viewed as causing side-effects and addiction, e.g., “there is a lot of stigma to taking medicine, especially by those in urban areas who have heard about pharmacological drugs” (participant, India). In South Africa and Pakistan, participants suggested the inclusion of personalised support related to religion and God, where this was considered helpful by the vast majority of participants, whereas this was rarely mentioned in other countries. In the UK, more contextual aspects of mental health were suggested as additions (e.g., promoting equity, access to quality health services), whereas participants in other countries less frequently mentioned contextual factors. Finally, in Pakistan, “increased financial resources via cash transfer” and “better urban access to green spaces” were difficult for participants to understand, as welfare agencies may not be readily accessible, and green spaces are limited.

We also found that words such as “better”, “positive”, “negative”, “improved” or “reduced” may not always be appropriate. Young people in particular raised questions such as “who decides whether an activity is positive or negative?” or “who decides whether a young person needs to develop better thought processes?”. Individual circumstances may strongly influence whether an activity or psychosocial state needs to be improved or reduced, and how the individual interprets it. For example, participants in Portugal said that within their context, young people need to be a little bit more perfectionistic, particularly when they go through depressive states.

### Micro Level

#### Understanding and Accepting Yourself

Developing insights about one’s own thinking and experiences, self-compassion, and positive self-regard were considered important. This aligns with previous work on self-compassion, (e.g., Neff, 2011; Neff & McGehee, 2010). Thus, young people described the importance of recognising the difficulties being experienced, or *acknowledging and accepting how things are,* as being a crucial first step to then being able to seek and access support, e.g., “accepting what is happening with oneself is a step to get better […] when there is not much self acceptance it will be hard to look for help and get the tools to change” (participant, Portugal).

A range of adaptive emotion regulation strategies were discussed, including *understanding, expressing and dealing with emotions*. Mirroring acknowledging and accepting life circumstances and events (also see previous theme)*, understanding and accepting emotions* was described by both groups as important, as appreciating the fluctuating nature of emotions may help reduce the experience of pressure to feel “well” all of the time. That is, young participants appeared to conceptualise wellbeing as an adaptive state rather than a reflection of “happiness”.

Accessing a range of *helpful thinking styles:* adaptive cognitive styles and strategies, was discussed in a similar context, and supports previous research (Lau et al., 2021). There were mixed views regarding *learning to manage perfectionism.* Generally, professionals described perfectionism often being imposed by unrealistic idealistic models through social media, thus being unhelpful and potentially contributing to experiencing depression and/or anxiety if these “perfect” standards could not be reached.

Young people suggested, however, that perfectionism could act as a helpful motivator for some people in certain circumstances, e.g., “we do not need to reduce our perfectionism unless that brings negative implications for our lives, those repercussions are what we need to reduce" (participant, Portugal). Both participant groups described a similar nuance in relation to *developing the ability to manage and face your fears,* where controlled exposure was described as helpful for anxiety, particularly activities central to functioning.

Different ways an individual may experience meaning and direction to their life were discussed, for example, *faith, spirituality and meaning of life*. A *sense of purpose* and the *ability to be optimistic and hopeful about the future* (e.g., through *goal-setting)* were described by both groups as important aspects of personalised support, bringing a sense of purpose. *Practising religious activities (religiosity) and religious or spiritual beliefs* were described as benefiting a range of areas, including social connections and relaxation, such as by *being connected to a higher power, e.g., “religious practices and faith gives power to majority of our people” (participant, Pakistan).* This finding aligns with previous research, which identified spirituality supporting meaning-making, identity and coping (Milner et al., 2019).

The importance of engaging in *regular activities and hobbies* that benefit self-care were described by young people as providing a constructive source of distraction, e.g., *engaging with creative activities:, “if we can enjoy being alone, doing something that we like hobby, sports. In my case, there was a time when I could not be alone because of thoughts […]. Now I can spend more time with myself and that is really important to me, so I can focus on what I really need (participant, Portugal).* Evidence in support of the benefits of young people engaging in creative activities include building resilience, improving problem-solving, and enhancing prosocial behaviour (Easwaran et al., 2021).

### Meso Level

Relational issues and *understanding and acceptance from others* were discussed. A trusting and authentic relationship between the young person and their social group, which recognises the individual as an agentic actor, was consistently reported by both groups, with *feeling understood and accepted by family and friends* being important for informal support but also for validating a young person’s experiences, e.g., *“[…] it's important to feel peace in the family. […] It can create a sense of trust. If he feels peaceful, then he does not have anxiety, he starts to think more rationally”* (participant, Turkey).

The importance of meaningful *social connections and relationships*, and reduced loneliness, at a range of levels, through social relationships, neighbourhood cohesion, and community engagement was discussed, e.g.,* “it's saying we need to feel like we have a sense of connection with others. And I guess different people get that through different ways. […] it could be like within those relationships you already have, just being more connected and there” *(participant, UK). This aligns with previous research, particularly focused on marginalised groups of young people (Sapiro & Ward, 2020). Both groups highlighted the crucial role of *relationships with family* for support.

Supporting young people to be actively involved in decisions and actions regarding their own mental health, their mental health care, and wider systemic and social changes was considered important. Both groups described *empowering young people to manage their health and the support received* as a key facilitator of effective support for depression and/or anxiety. Empowering young people to manage their health and support and their lives more generally was described as a beneficial and bi-directional relationship. In addition, *access to advocacy and representation* was described as important in enabling young people to have a voice in their care and their lives, which echoes previous findings on the benefits of advocacy for young people (Colucci et al., 2015; Ridley et al., 2018; Thomas et al., 2017), e.g., *“I'm thinking about situations where young people might not feel listened to at all and then, that might lead to issues. Like increased access to advocacy”* (participant, UK).

Individual and collective *knowledge about mental health and how to access help* to facilitate help-seeking, engagement with support, and also having a secure relational foundation to feel safe whilst doing so, was discussed (see also: understanding and acceptance from others), e.g., “I* think education about de-stigmatising these things – I don’t think I saw it once, but I think that’s also really important. Because no matter how much you try and change the system, if you don’t change people’s views it won’t really get better”* (participant, UK). This finding is consistent with previous research identifying social support and encouragement from others as key facilitators to help-seeking (Gulliver et al., 2010). Psychoeducation was described as necessary for young people, parents and carers, schools, and the community. Improved knowledge, in turn, was described as enabling young people to engage with formal mental health support, including professional help and pharmacological treatment, so that they could become more familiar with these concepts from the outset. Improved knowledge was described as particularly important to address stigma.

### Macro Level

The socio-economic infrastructure is required to support young people’s mental health, beginning with meeting basic needs, safety, welfare, and access to services. *Increased financial resources and financial stability (e.g., cash transfer)* was described as important but nonetheless, one that does not necessarily directly impact youth mental health, e.g.,* “It is not that money brings happiness, but if you have the resources to support yourself, eat well, do activities, it helps a lot. So having no income, certainly has an influence”* (participant, Brazil). However, previous research has found a significant positive impact of cash transfers on at least one mental health outcome in children and young people (Zimmerman et al., 2021). Another limitation raised was about the sustainability of, for example, providing ad hoc cash transfers as an ongoing model. Similarly, both stakeholder groups described the importance of *accessing information for financial support and better management of money* to empower young people to actively manage their own lives (see also: empowerment).

*Promoting equity and decreasing discrimination and social injustice* was described as important to address the social determinants of anxiety and/or depression (see Vargas et al., 2020). Recognising these determinants was perceived as insufficient without corresponding action to redress them, e.g., "*there maybe needs to be something about systematic stuff, in the wider society. Like better urban access to green spaces, and if we're including that, that's to do with the more social side of things. Then, we can include poverty and discrimination […] it's not just about understanding and awareness, it's about actually changing those things. We're not individuals, but we're living in a bigger system which affects us"* (participant, UK).

*Access to a workforce that represents young people and their communities,* was described as important to facilitate the effectiveness of other aspects of personalised support. This includes feeling understood in terms of how someone’s identity impacts the way they understand and talk about mental health.

## Discussion

The aim of the present research was to conduct an international qualitative study of the views of young people with lived experience and professionals on proposed aspects of personalised support to prevent and treat anxiety and/or depression. Through participatory action focus groups, eleven themes were developed as a way to organise aspects of personalised support for young people, which addresses the first research question: “what aspects of personalised support do young people with lived experience and professionals view as effective for young people experiencing anxiety and/or depression?”.

Overall, although country-specific differences were evident, individual preferences were considered stronger, supporting the view that support should be personalised and tailored to the needs of the individual young person. This partially supports the second research question: “are there country-specific differences in the aspects of personalised support that young people with lived experience and professionals view as effective for young people experiencing anxiety and/or depression?”. Systems of support that are not tailored to individual needs lead to reduced access for many young people. The nature of historically built systems of support that align with White Western concepts relating to the drivers of mental health difficulties, the understanding of these difficulties and therefore the best ways to support young people exclude marginalised and minoritised groups from both accessing and receiving quality care (see, Bansal et al., 2022). While the present researchers were Europe-based, the international nature of this research goes some way towards including a diversity of experiences and thought into research on the effective aspects of personalised support for young people. Further, the present research offers an evidence base that supports how mental health can be perceived and communicated about with young people, e.g., in a prevention and early intervention sense in schools and community settings, as well as within specialised support settings, increasing knowledge and increasing the opportunities for much needed support for more young people.

This research builds on the existing theory and knowledge base about “what works” for young people’s mental health, by establishing young people’s voices and perspectives. Specifically, the findings suggest that a range of cognitive, emotional, behavioural, relational, and systemic factors contribute to the prevention and treatment of mental health difficulties, which supports previous research (e.g., Stirling et al., 2015). While the present research has direct implications that could help inform clinical decisions, further local research is needed to supplement this work, to explore precisely how personalised support can be effectively incorporated into routine practice, taking into consideration the relevant policies systems and practices both across and within countries. The international nature of our research highlighted the need for considerations to be made regarding the way potentially effective aspects of personalised support are discussed. Each culture provides different contexts and experiences of social reality can promote different interpretation, with particular differences between participants across countries. Language is central to qualitative approaches; therefore, it is important that translation into all languages is as accurate as possible. The linguistic specificities and the difficulty of semantic understanding seems to be a constraint identified by peer advisors and peer researchers. Some words do not have a literal translation into the participants’ local language, hence requiring a deeper explanation. Cultural contexts also led to a variation in perceived relevance from country to country. For example, learning to manage perfectionism was listed as an aspect that could reduce anxiety and/or depression, but young people in Portugal felt that perfectionism could in fact be beneficial during times of depression as it promotes higher level functioning. This is in line with previous findings that, although self-critical perfectionism may lead to distress and mental health problems, positive adaptive perfectionism can lead to achievement and positive outcomes for young people (Morris & Lomax, 2014). Other cultural contexts may only view perfectionism as something that can be self-critical, and therefore needs to be managed so that it does not lead to distress. This further highlights the necessity of personalising care by tailoring support to the young person’s context and creating space for working in such a way that may help to promote change and flexibility. This supports previous research demonstrating that young people are more likely to access mental health support if they have some previous knowledge about mental health and the support available to them (Rickwood et al., 2007).

Further, young people also strongly indicated that the choice of engaging (or not) in some of the aspects of personalised support should sit with young people, rather than with mental health professionals, which is consistent with other research findings that health professionals should involve young people in shared decision-making as an approach to personalised support (Krause et al., 2023). This is a key finding for professionals working to support young people’s mental health. Individual participants reported clear preferences for personalisation regarding the management of their own anxiety and/or depression. These preferences can be categorised as individual, experience-based, and country-specific preferences. For example, personal preferences were found to be influenced by socioeconomic and educational frameworks, along with young people’s experiences and developmental state. We found that specific circumstances including experiencing financial difficulties can impact young people's choices regarding helpful aspects of personalised support. Some young people value change and psychological wellbeing over financial aspects, and those with economic stability tend to consider that it may not be relevant for their mental health. While we did not incorporate parent and carer perspectives in our study, parents and carers also influence young people’s access to mental health support, which may be influenced by factors such as financial difficulties. These can be prioritised over mental health difficulties, particularly in countries where healthcare is not free at the point of access (see e.g., RothÌ & Leavey, 2006). Future research should explore the combination of factors in more depth, to better understand the interconnections between them.

A central determinant of our research was working with young people in a meaningful way, both as participants of the research, and as key members of the research team. This allowed us to gain unique insights into the opinions of young people that are not necessarily part of the dominant research view. For example, young people shared their thoughts on how controlled exposure can help with developing the ability to manage and face your fears, and, although this has been supported by preliminary findings, challenges with the consistency of previous research mean that the ability to draw strong conclusions is limited (Plaisted et al., 2021). Regardless of the quality of previous research, these are still important insights to consider as it could suggest some views have not been adequately explored before, and so need to be explored more rigorously in future studies. Young people’s voices can therefore help to inform future research directions. Young people could also be valuable ambassadors, co-facilitators, mentors or educators in helping their peers (or, indeed, younger children) in their schools and communities to access and benefit from the findings of this study. We should thus aim to better understand, formulate and test these roles in different contexts. There is already evidence of young peer mentors in the international mental health context (e.g., see systematic reviews: Douglas et al., 2018; Fortuna et al., 2020). As such, our recommendations are in line with other initiatives, while adding further guidance about how peer mentors can interact with their mentees. The actual work between mentors and mentees differs between interventions and therefore it may be beneficial for young peer mentors to discuss with their mentees suggested personal facilitators of change that they have co-produced themselves.

There are also several worldwide youth mental health advocacy programmes and youth-focused awareness campaigns that seek to reduce stigma and support youth mental health, e.g., international youth Mental Health First Aid (Kitchener & Jorm, 2008), Headspace day (Rickwood et al., 2014) and Batyr (Lindstrom et al., 2021) in Australia, Jack.org in Canada (Jack.org, n.d.) and YoungMinds in the UK (YoungMinds., 2020). Young people, families and communities need to be given the required tools to ensure interventions are tailored to the young person’s needs and that they are able to effectively navigate support for depression and anxiety difficulties; incorporation into these initiatives is a good starting point.

When working with young people, personalisation of mental health care is crucial to the process of recovery. Our recommendation is to ensure the language and medium used to describe the aspects of personalised support can be understood by young people and professionals from different contexts, including marginalised and minoritised groups and communities. This includes, but is not limited to, translation into different languages, and providing explanations for the meaning behind certain aspects of personalised support, where necessary. As individual preferences may be more relevant than country-specific preferences, we suggest using one list of aspects of personalised support internationally, with the option for young people to make the list tailored to their preferences. In addition, individual young people, rather than their family or mental health professionals, should be able to decide which mechanism(s) they prioritise based on what is most important to them (with guidance from adults, where appropriate). Mental health services may need support with adapting practices in some contexts in order to support young people in this person-centred way, and specific training on how to facilitate the shared decision-making process may also be beneficial for some mental health professionals. At a higher level, government mental health strategies should incorporate ideas about personalised care, as has already been done in countries such as the UK and Australia, and work towards improving the organisation of resources to facilitate this approach, including in LMICs where resources may be limited, and services may already be under pressure from competing health priorities. Finally, young people need to be supported not only to access and understand the aspects of personalised support available to them, but also how to use and implement them, especially at times when engagement with mechanisms may be most challenging (and most important).

## Strengths and Limitations

The study involved a range of participants from high- and low- to middle-income countries and centralised the voice of young people. The use of young people with lived experience arguably grants our findings greater relevance and usefulness, by directly addressing the needs of those who have accessed mental health services (or may do so in the future). This could also help to close the gap between knowledge and practice in mental health research and empower both youth and professional participants (Ghisoni et al., 2017; Lee et al., 2023; McCabe et al., 2023). However, there were practical limitations regarding accessing countries, meaning that no participants from North and Central America, Oceania, Australia, or East and South-East Asia were involved. The international nature of the study could be improved in future by working to address these gaps. Similarly, the majority of young people involved were students enrolled in university or higher education, with some currently training as health professionals, and so were part of a unique demographic and may have had more awareness of their mental health needs. This meant that the voices of young people who are in more disadvantaged or less educated groups were missing. Further, while the research on personalised support lends itself to prevention and early intervention, the research findings lean towards specialised support, which is due in part to the emphasis on anxiety and depression, and thus the participants who were recruited due to their lived, or professional, experience of these specific mental health difficulties. Considerations for further research could be to explore how the key mechanisms can be applied to representative general populations to ensure the benefit of personalised support for all.

The diversity of participants was also limited in terms of gender identity, with around 70% of participants identifying as women. It is important to be inclusive of gender diverse young people, and young men, to ensure their experiences inform mental health support, especially as they are less likely to receive such support (Brown et al., 2019; Snow et al., 2019). Although this gender bias may not have had an impact on the general trends in discussion, future research building on the findings here may benefit from employing recruitment strategies that aim to increase the involvement of these underrepresented groups in the discussion around mental health. Also, the 26 personalisation aspects put forward by the Wellcome Trust provided a useful conceptual context for the study. A limitation of this framework was that it had not been empirically tested at this stage. While the 26 personalization aspects were originally suggested to the Wellcome Trust by researchers, a truly data driven approach in this research might have produced different findings.

## Conclusions

An important recommendation arising from this study is the need for young people, families, professionals, and communities to have access to tools and resources to be able to support young people experiencing anxiety and/or depression, and in some instances, assist with prevention. The field of implementation science suggests that contextual factors are most important when considering the journey from research into practice (Bauer & Kirchner, 2020). These factors are multifaceted and include the individual, staff, and organisational levels, as well as the financial and political environments within which support is provided, and the broader societal context (Glasgow et al., 2012). Further consideration should be given to the interfaces between individual preferences and these contextual factors, when considering the personalisation of care. The personal freedom of young people to integrate and accept their experiences is also a relevant factor that should be considered in the design of interventions and services. Finally, since our study, many of the identified factors have been the focus of literature reviews, generating some promising, but overall mixed, results. Therefore, more research is needed into the specific mechanisms of change for young people experiencing anxiety and/or depression (see Wellcome Trust, 2021).

## Data Availability

Qualitative data are not available for sharing, given the risks of identification.
